# Achieving arithmetic learning in honeybees and examining how individuals learn

**DOI:** 10.1080/19420889.2019.1678452

**Published:** 2019-10-15

**Authors:** Scarlett R. Howard, Aurore Avarguès-Weber, Jair E. Garcia, Andrew D. Greentree, Adrian G. Dyer

**Affiliations:** a Centre de Recherches sur la Cognition Animale (CRCA), Centre de Biologie Intégrative (CBI), Université de Toulouse, CNRS, UPS, Toulouse, France; b Bio-inspired Digital Sensing (BIDS) Lab, School of Media and Communication, RMIT University, Melbourne, Australia; c ARC Centre of Excellence for Nanoscale BioPhotonics, School of Science, RMIT University, Melbourne, Australia; d Department of Physiology, Monash University, Clayton, Australia

**Keywords:** Addition, arithmetic, learning, numeric cognition, subtraction

## Abstract

In recent years honeybees have demonstrated intriguing numerical capacities, leading to the recent discovery of their ability to perform simple arithmetic by learning to add or subtract ‘one’ using symbolic representations of operators. When training an insect with a miniature brain containing less than one million neurons to understand a conceptual rule, the procedure is of vital importance. We explain in detail the controls and process of designing an experiment to test for complex behaviors in a relatively simple brained animal. Furthermore, we will discuss the finding that individual honeybees do not demonstrate a consistent learning scenario when trained to perform the same tasks, rather they appear to acquire arithmetic rules through individual processes.

Arithmetic, such as addition and subtraction, is considered a complex task to perform due to the simultaneous requirements of both short-term/working memory and long-term memory [1]. Working (short-term) memory is used to manage the numerical values (quantities) while performing the operation, and long-term memory is used to store the rules for adding or subtracting. Arithmetic has so far been demonstrated by non-human animal species including various primates: vervet monkeys [1], rhesus monkeys [2], chimpanzees [3,4], and orang-utans [5], birds: a single African gray parrot [6,7] and pigeons [8], and now insects: honeybees [9]. The wide range of taxa that can perform addition and/or subtraction suggests that more animal species could share a capacity to learn arithmetic if given the correct opportunity.

Honeybees are capable of learning rules such as size discrimination [10,11], ‘above/below’ [12], and ‘same’/’different’ [13], among many others. The honeybee is emerging as an ideal invertebrate with which to examine numerical abilities [14,15]. Honeybees have displayed the capacity to learn and perform a number of numerical abilities such as landmark counting [16–19], quantity matching [20], discriminating ‘more’ vs. ‘less’ items [21], using absolute number discrimination [22], understanding the quantitative value of ‘zero’ as a number below 1 [21], learning to match characters with quantities (symbolic representation of quantity) [23], and now, simple arithmetic [9]. We recently demonstrated, that with careful training procedures, honeybees could learn to use color as a prompt (symbolic cue) to add or subtract one item from an array of objects within a stimulus presented to them within a Y-maze. Bees were trained using a procedure known as delayed-matching-to-sample (DMTS; Figure 1). This process involves an individual viewing a stimulus in isolation, then viewing secondary stimulus/stimuli separated in time (i.e., delayed and thus requiring memory) from the initially viewed sample. In some cases, the individual bee needs to match the initial sample with one of the subsequent options, such as in ‘same’ vs. ‘different’ tasks [13,20], in other experiments, individuals need to use the initial sample as a prompt to determine which is the correct option [9,23] (Figures 1, 2).

**Figure 1.**
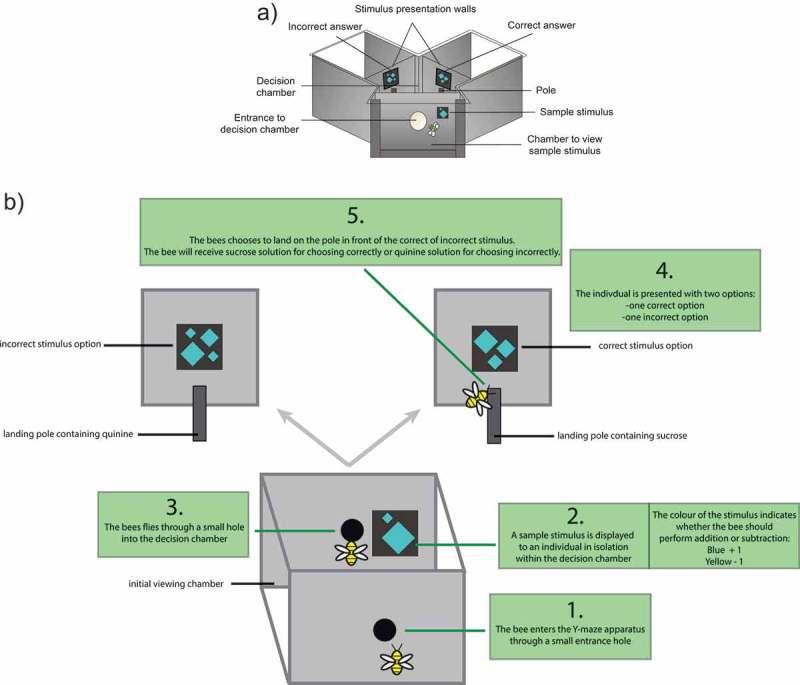
A schematic showing the sequential process of individual bees entering the Y-maze apparatus, viewing the initial sample stimulus, flying into the decision chamber, and then making a choice between the correct and incorrect stimuli. (a) The overall view of the Y-maze. The positions of the correct and incorrect options (left or right arm of the maze) were changed pseudo-randomly to avoid bees acquiring a side preference (preference to always fly to the left or right). (b) An example of the process of using the Y-maze by a bee in the ‘addition’ trials.

**Figure 2.**
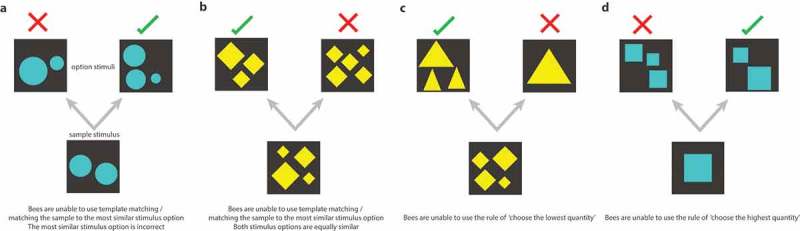
Examples of trials in which bees could not employ the rules of ‘choose the most similar stimulus’ (a–b), ‘choose the lowest quantity’ (c), or ‘choose the highest quantity’ (d). Stimuli were never shown to a bee more than once in training, and in testing stimuli were of a novel shape and pattern, thus bees could not use associative mechanisms to solve the task.

Using this DMTS procedure (Figure 1), we initially presented bees with a card containing one to five elements (the sample). For the experiments, the elements were either blue or yellow, and could be squares, diamonds, circles, or triangle shapes. After viewing this initial array of elements, bees would fly into a ‘decision chamber’ where they were presented with two options. If the initial array of elements had been blue, bees would need to choose the array in the decision chamber which contained one more element then the initial array to receive a reward of sucrose solution. However, if the array of elements had been colored yellow, the bee would need to select the option in the decision chamber which was one less than the initial array. Honeybees received a reward of sucrose solution to reinforce a correct choice and an aversive outcome of quinine solution to reinforce an incorrect solution [24]. During testing, bees were presented with a completely novel sample stimulus, in terms of quantity, shape of the objects displayed and spatial organization, thus bees would be unable to use an associative mechanism (responding to a particular stimulus that have been associated with reward beforehand) to solve the test tasks.

To increase the complexity of the task, the incorrect option could be higher, lower, or the same as the initial array of elements. The stimuli were also of an equal element surface area (Figure 2). This meant that bees were unable to employ a simple low-level strategy to find the correct option such as ‘choose the most similar array to the sample’ because if they chose the option visually closest to the initial array it would be incorrect as the sample number would be incorrect regardless of whether the elements had been blue or yellow (Figure 2(a)). For example, if the number shown was three and the elements were yellow, the correct answer would be two. If the correct answer was shown against the incorrect answer of three, using the ‘choose the most similar array’ option for a bee would be easiest but would result in a punishment of quinine. This is how we were able to rule out bees using a simple retinotopic-template matching mechanism for solving the task [10,25] (Figure 2(a)). Additionally, if the incorrect answer had been four, then the correct and incorrect answers would be in competition for what was most similar to the initial array of elements, as both are one element different to the number three (Figure 2(b)). Bees were also unable to use a solution of ‘if yellow, go to the lowest number’ as in some cases the lowest number was incorrect (Figure 2(c)). For example, in a subtraction trial presenting a sample of three, where two is the correct answer and one element is the incorrect option, bees using a ‘choose the lowest number’ option would also be incorrect (Figure 2(c)). Thus, we ensured it was too difficult for bees to try to use a lower-level cue such as this. This control was also in place for bees when the array was blue, as they would not be able to employ a ‘choose the greatest quantity’ solution (Figure 2(d)). Thus we controlled for bees using rules such as ‘choose the most similar stimulus’, ‘if elements are yellow choose the lower number’, ‘if elements are blue choose the higher number’, and also ‘choose the option with the most similar surface area’. Therefore, bees demonstrated acquisition and extrapolation of a complex numerosity task, without using the simple cues described above.

The proportion of correct choices observed in our population of bees shows steady learning, from chance level (50%), up to 80% success probability after 100 trials, where we have averaged the performance of the 14 bees. This data is shown in Video 1. However, we wanted to understand this performance by analyzing the performance of each individual bee. We had initially suspected that there would be an ‘aha!’ moment indicating a definitive learning event [26] when each bee ‘understood’ the addition/subtraction rules. For example, a step function-like learning curve, where we observe bees switch from chance level to a consistently high rate of success. However detailed analysis of the individual bee performance could not substantiate this hypothesis.

To analyze individual bee performance and account for the assumed errors in performance, we performed a Bayesian analysis of the bee performance where we treated the bee performance as a random variable corresponding to the probability that she will achieve the correct solution in a given trial (Video 1). A value of 50% indicates chance performance, below 50% indicates a bias toward failure (which may be expected if an individual is applying incorrect rules when attempting the problem), above 50% indicates a bias toward success, and 100% indicates perfect success. By performing a Bayesian analysis, we can determine the probability of achieving a certain performance rate, as a function of number of trials, considering performance over a given set of trials, here chosen to be 10 trials (Video 1).

The results of this Bayesian analysis for 14 individuals are given in Video 1, along with the success or failure of each trial. The red line is the 10 trials running average of performance, and the color indicates the probability of a particular success probability. As can be seen, there is no evidence of a consistent ‘aha!’ moment [26] among individual bees, and the success probabilities as a function of trial seem to be very different from individual to individual. Variation in performance between individuals is known for honeybees [27,28], although it remains to be shown whether the difference in performance is due to individual variation, the difference in the pseudo-random ordering of the stimuli, or another currently unknown factor. Of related interest is the question of trying to determine what rules the bees are applying as they begin to learn and whether there might be some ‘optimal’ training set to achieve maximum performance in minimum number of trials. We were unable to address these questions due to constraints of time and number of individuals, but we believe these questions are fundamental to understanding the mechanisms by which bees can learn novel rules and may have wide ranging implications from neuroscience to artificial intelligence.

Our study demonstrates that bees are capable of learning and extrapolating simple arithmetic, specifically adding and subtracting one element from an array of elements. While we controlled for many factors and low-level cues in order to enable bees to learn simple addition and subtraction, we do not claim to fully demonstrate that honeybees are capable of more complex arithmetic. More research will be needed to determine if their abilities are limited to adding and subtracting by one, or if they could perform more complex addition and subtract involving higher numbers, multiple addends, multiplication, or division. However, the results of Howard et al. (2019) do show that an insect with a brain of less than one million neurons is capable of learning and applying complex numerical rules such as ‘add one’ and ‘minus one’, thus demonstrating that a miniature brain does not restrict cognitive capability to the extent that was previously thought [29]. The demonstration that honeybees can perform simple arithmetic using symbolic cues allows us to consider how far their numerical ability, and the numerical ability of other invertebrates, may reach.
