# Supplementary figures and images for: Achieving arithmetic learning in honeybees and examining how individuals learn
Source: Commun Integr Biol. 2019 Oct 15;12(1):166–70. doi: 10.1080/19420889.2019.1678452 (PMC6802933; doi:10.1080/19420889.2019.1678452)

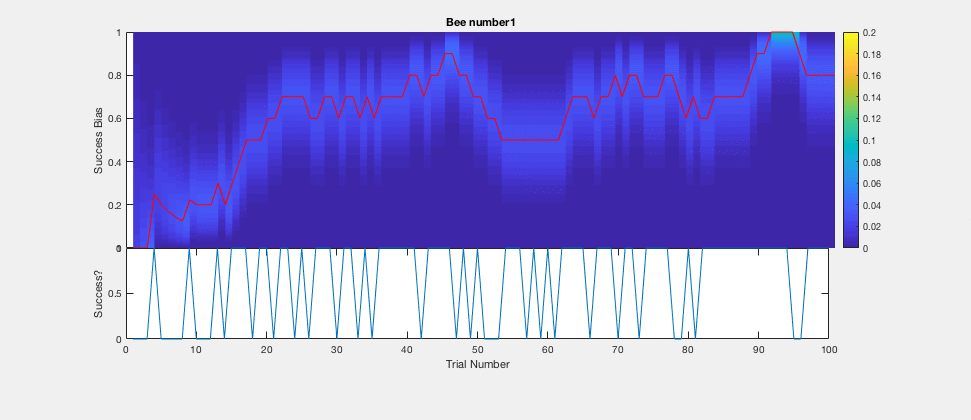

Supplement: Supplemental Material [file kcib-12-01-1678452-s001.gif]
